# History of Medical Delusions

**Published:** 1850-05

**Authors:** 


### ECLECTIC DEPARTMENT,

### AND SPIRIT OF THE MEDICAL PERIODICAL PRESS.

---

*History of Medical Delusions.*—The following sketch of *three,* among the many medical delusions famous in past history, is taken from an interesting Address delivered before the *Alabama State Medical Association,* Dec. 10, 1849, by William O. Baldwin, M. D., and published by order of the Association. The Author introduces this sketch by way of introduction to some able remarks on the medical delusions of the present day, and particularly the delusion which, in many respects, resembles each of the three described, to wit: *Homœopathy.* The historical facts presented in the sketch may not be new to many of our readers. They are probably contained (as the Author intimates), in the treatise by Dr. Holmes on *Homœopathy and its kindred delusions,*—a work which we have not yet had the pleasure of reading. As illustrative of the facility with which absurd doctrines relating to diseases and remedies may be imposed upon popular credulity—a facility scarcely less at this, than at any past period, and which bids fair to triumph over all the lessons of experience—the three delusions described in the following extract are interesting; and, we have thought, will prove acceptable to our readers.—[Editor Buffalo Medical Journal.

The Royal Gift of Healing was the birth-right of Kings, and was practiced for the cure of Scrofula, or King's Evil. It was first practiced by Edward the Confessor, King of England, who ascended the throne in 1014. This power, it was admitted, extended to the potentates of other nations, but only such as weie connected with the royal family of England. The French, however, contended that this sacred privilege belonged to their Kings exclusively. Certain it is that it was practiced by the sovereigns of both nations over a period of nearly 790 years. William III discontinued the practice, but Anne resumed it.

There was but little ceremony observed on the occasion. On the patient's being presented, the sovereign touched him with his hand, and then hung a piece of gold or silver around his neck. This completed the treatment, and generally the cure. In proof of the latter, abundant evidence like the following can be adduced :—

Jeremy Collier, when speaking of Edward the Confessor, in his Ecclesiastical History of Great Britain, says, "that this prince cured the King's Evil is beyond dispute : * * * * * To dispute the matter of fact is to go to the excesses of skepticism, to deny our senses, and be incredulous even to ridiculousness." Bradwardine, Archbishop of Canterberry, who lived during the reign of Edward III and Richard II, in speaking of this practice, says, " Whoever thou art, O Christian, who denyest miracles, come and be an eye-witness of their truth." Sir John Fortescue, Lord Chief Justice of the King's Bench during the reign of Henry IV, and also Chancellor to Henry VI, says, " the Kings of England, at the time of unction, receive such a divine power, that by the touch of their hands they can cleanse and cure those who are otherwise considered incurable, of a certain disease, commonly called the King's Evil." The Rev. Dr. Wm. Tooker, who was chaplain to Queen Elizabeth, Canon of Exeter and Dean of Litchfield, wrote a history and defence of this power, but confined it to the English sovereigns, and extols very highly the powers of the Queen. Badger, in a report of cases of the King's Evil which were cured by the imposition of royal hands, says of Charles I, that he " excelled all his predecessors in the divine gift ; for it is manifest, beyond all contradiction, that he not only cured by his sacred touch," &c.

So important was this portion of the King's duty considered, that frequent proclamations and special bulletins were issued in regard to it ; and the practice of, as well as the confidence in this mode of curing the disease, was so general, that it became a work of exceeding labor to them, and they consequently generally gave notice that they would exercise this power on appointed days. In order to prevent people from applying who were not really subjects of disease, applicants were required to bring certificates from one of the surgeons, who had been appointed for the purpose of examining them. The surgeons also had their regular hours for making these examinations, and so great was the effort to get to their offices on such occasions, that six or seven persons were known to have been crushed to death, in the scramble, in one day. In the diary of Bishop Cartright, he says, in speaking of James II, " I attended his majesty's levee : from whence, at nine o'clock, I attended him in his choir, where he healed 350 persons" ; and it is stated of one of the French Kings, Louis XIV, that he touched and cured 1600 in one day. The number of cases touched by Charles II, of England, according to a register kept by the sergeant and keeper of his closet, amounted to 92,107, and this, too, in the space of twenty years. Judging from the comparative infrequency of the disease in the present age, one would be inclined to think that this comprehended not only all of the real, as well as imaginary cases of Scrofula which occurred in his raalms during this period, but also all that occurred in the world. At all events it is a pretty good evidence of the great popularity of the practice. Indeed, so overwhelming was the cry of " cures," " cures" and the number of cases reported as such, that we find many of the physicians withdrawing opposition to the practice, and even prescribing this as a means of cure for their patients. Perhaps the fear of royal displeasure and popular prejudice, may have extorted this acknowledgment from them. In any event it shows the mass and plausibility of the evidence which has been accumulated in its favor. William Cloves, surgeon to Queen Elizabeth, in a treatise on Scrofula, says, " it is a disease repugnant to nature : which grievous malady is known to be miraculously cured and healed by the sacred hands of the Queen's most royal majesty, even by divine interposition and wonderful works of God, above man's skill, art and expectation. Through whose princely clemency, a mighty number of her majesty's most loyal subjects, and also many strangers borne, are daily cured and healed, which otherwise would most miserably have perished."

Wiseman, a surgeon and writer of some distinction, in speaking of the King's Evil, says, "I myself have been a frequent eye-witness of many hundreds of cures performed by his majesty's touch alone, without any assistance of chirurgery : and those, many of them, such as had tyred out the endeavours of able chirurgeons before they came thither. It were endless to recite what I myself have seen, and what I received acknowledgments of by letter, not only from the several parts of this nation, but also from Ireland, Scotland, Jersey and Guernsey. * * * * I must confess that what I write will do little more than show the weakness of our ability when compared with his majesty's who cureth more in one year than all the chirurgeons of London have done in an age."

Evidences of this kind could be multiplied without number, but it is thought that enough has been said to prove that this means of treating Scrofula obtained a high reputation by the number of cures which it was said to have performed. The practice continued for a considerable portion of the 18th century, and Dr. Samuel Johnson, known to you all as a distinguished lexicographer and moralist, was "cured" of Scrofula, it was said, when about five years of age, by the imposition of the hands of Queen Anne. On the same day that he was "cured," she touched two hundred other persons for the same disease.

It is strange, however, when stated in connection with the general resort to this *sovereign* remedy, and in the face of all these repeated cures, that more persons died of Scrofula during the reign of Charles II, (who practiced this "divine gift" more extensively than any other monarch,) than in any period embracing the same length of time.

The *Sympathetical Cures* were practiced more extensively in the reigns of James I and Charles I. There were two different modes of conducting this practice. One was the Sympathetic Powder of Sir Kenelem Digby, the secret of which he obtained from a Carmelite Friar, who had been an extensive traveller, and obtained his knowledge of this wonderful remedy during his peregrinations in the .East. This Friar was much disposed to keep his knowledge a secret still, having resisted the entreaties of the Grand Duke of Florence ; but the versatile Knight succeeded in bringing him under obligations which called so strongly upon his gratitude that he revealed his secret to him. There was about as much hocus-pocus observed in the preparation of this powder, as there is in the trituration of some of those of the present day. It was to be crystalized, dissolved, filtered, and re-crystalized, and this process several times repeated. It was thought necessary to expose it to the action of the sun 365 days, much care being taken, during this time, to secure to each crystal the action of the rays, and the processes of triturating, dissolving, and re-crystalizing, was continued in rapid succession ; and, at last it came out what it originally was—*Blue Vitriol.*

This powder was used for the purpose of curing wounds, but instead of being applied to the wound itself, a portion of it was dissolved in water and any garment, or substance which had been stained with the blood which had escaped from it, was steeped in the solution, whilst the wound was dressed and left seven days to Nature, and the influence which this powder could exert on it at a distance.

The following is a remarkable cure which it is said to have performed, and which established the practice in England: "Mr. James Howel, a gentleman celebrated by his 'Dendrologia,' and other works, in endeavoring to part two of his friends in a duel, received a severe wound of his hand. Alarmed at this accident, one of the combatants bound up the cut with his garter, took him home, and sent for assistance. The King, upon hearing of the event, sent one of his own surgeons to attend him ; but, as in the course of four or five days, the wound was not recovering very favorably, he made application to Sir Kenelm Digby, of whose knowledge regarding some extraordinary remedies for the healing of wounds he had become apprized. Sir Kenelm first inquired whether he possessed anything that had the blood upon it, upon which Mr. Howel immediately named the garter with which his hand had been bound, which was accordingly sent for. A basin of water being brought, Sir Kenelm put into it a handful of powder of vitriol, and dissolved it therein. He then took the bloody garter and immersed it in the fluid, while Mr. H. stood conversing with a gentleman in the corner of the room ; but he suddenly started, and upon being asked the reason, replied that he had lost all pain—that a pleasing kind of freshness, as it were a wet cold napkin, had passed over his hand, and that the inflamation, which before had been so tormenting, had vanished. He was then advised to lay aside all his plasters, to keep the wound clean, and in a moderate temperature. After dinner the garter was taken out of the basin and placed to dry before a large fire : but no sooner was this done than Mr. H.'s servant came running to Sir Kenelm, to say that her master's hand had again inflamed, and that it was as bad as before: whereupon the garter was again placed into the liquid, and, before the return of the servant, all was well and easy again. In the course of five or six days the wound was cicatrized, and a cure performed." Through the influence of James I, and his surgeon Dr. Mayerne, known to commentators as the Dr. Caius of Shakspeare, the practice was extended throughout Great Britain and France, so that not only Sovereigns, Princes, Knights, and *Court* Physicians practiced it, but there was scarcely a village barber who was not prepared to treat wounds after this fashion.

Sir Kenelm Digby, in a lengthy discourse before an audience composed of noblemen, and other distinguished personages, at Montpelier, attempted to explain the rationale of its operation upon philosophic principles. His views in relation to the emanation of light, the impinging rays of the sun, the formation of wind, &c., as bearing on his doctrines of the Sympathetical Cure, I am sorry I have not space to introduce, as I am sure, that like some of the propositions and arguments of Homœopathy, if they possess no other merit, their subtle and refined ridiculousness, and want of common sense, would amuse you.

The *Unguentum Armarium*, or Weapon Ointment, was also used for the cure of wounds, but, like the Sympathetic Powder, instead of benig applied to the wound, the instrument which inflicted it was anointed, carefully wrapped up, placed away, and dressed anew whenever the wound became painful. If the sword, or instrument with which the wound was made, could be found, it was thought all sufficient to make one like it, which was treated as if it was the original.

The ungents used for this purpose were made from human fat and blood, mummy, moss obtained from dead men's skulls, bull's blood, &c. A serious and animated discussion was for a long time kept up in the Sympathetic School, in consequence of a division among its members as to "whether it was necessary that the moss should grow absolutely in the skull of a thief who had hung on the gallows, and whether the ointment, while compounding, was to be stirred with a murderer's knife?"

A case is reported, in which it is said that Lord Gillbourne, an English nobleman, performed a wonderful cu e upon a carpenter, who had severely wounded himself with an axe. "The axe, bespattered with blood, was sent for, besmeared with an ointment, wrapped up warmly, and carefully hung up in a closet. The carpenter was immediately relieved, and all went on well for some time, when, however, the wound became exceedingly painful, and, upon resorting to his lordship, it was ascertained that the axe had fallen from the nail by which it was suspended, and thereby became uncovered!" This was called the cure by the dry way, in contradistinction to the Sympathetic *Powder*, which was called the cure by the wet way.

There is no want of evidence to prove that this practice was extensively resorted to, and performed many very astonishing cures, not only in the treatment of wounds in the human subject, but also in those of the lower order of animals. Lord Bacon alludes to it in his Natural History, and speaks of the high testimonials in its favor. Sir Walter Scott, in the Lay of the Last Minstrel, introduces the practice thus :—

> "But she has ta'en the broken lance,
> And washed it from the clotted gore,
> And salved the splinter o'er and o'er,
> William of Delorain in trance.
> Whene'er she turned it round and round,
> Twisted, as if she galled his wound;
> Then to her maiden she did say
> That he should be whole man and sound."

Dryden makes Ariel say, when speaking in reference to Hippolito's wound :—

> "Anoint the sword which pierced him with this
> Weapon salve, and wrap it close from air,
> 'Till I have time to visit it again."

*Perkinism, or Metallic Tractors,* was a true-born Yankee, and I am proud to say, I believe it is the only thing of the kind that we ever floated upon our transatlantic brethren. Dr. Elisha Perkins, a native of Norwich, Connecticut, made known the discovery of a remedy for the cure of Rheumatism, inflammations, and various other complaints, which he termed the "Matallic Tractors," and which also bore the name of the inventor—"Perkinism." This great discovery (!) was made shortly after Galvani had completed his experiments and dircoveries in relation to the effect of two different metals in producing muscular contractions, when applied in contact, which were published to the world, for the first time, about the year 1791, and from which it is snpposed Perkins borrowed the idea upon which he founded his system.

This system consisted of two small bits of metal, one iron and the other brass, of the same shape and dimensions, about three inches long, pointed at one end and blount at the other. These were to be drawn over the diseased organ for about twenty minutes, and the cure, as many are willing to vouch, followed in a very few minutes after. The inventor took out a patent for the discovery, was expelled from the Medical Society to which he belonged in consequence, and very soon obtained high evidences of the value and success of the practice by a number of cures which were affirmed by clergymen, members of Congress, &c., who also very modestly gave it as their opinion that it was the most important discovery ever made in the Medical science—surpassing even those of Jenner and Harvey. Thus panoplied, in two years they had crossed the Atlantic. In Copenhagen they were employed at the Royal Hospital, and a large volume was published in the Danish language, containing accounts of experiments with them, and, in illustration, a vast number of cases in which they had performed cures. Among the highly educated and intelligent population of London they received much favor, and by them a Perkinean Institution was founded, and the distinguished Lord Rivers elected its President, and eleven other gentlemen, scarcely less distinguished, were elected Vice Presidents. The transactions of this Society were regularly published, and the triumphs of the system celebrated by public banquets.

Among those worthies who sent their evidence across the water, the names of thirteen clergymen, besides members of Congress, and other functionaries of minor importance appear. A "Perkinistic Committee" in Great Britain, in making their report, stated—"Mr. Perkins has annually laid before the public a large collection of new cases, communicated to them for that purpose by disinterested and intelligent characters, from almost every quarter of Great Britain. In regard to the competency of these vouchers, it will be sufficient simply to state that, amongst others whose names have been attached to their communications, are eight Professors in four different Universities, twenty-one regular Physicians, nineteen Surgeons, thirty Clergymen, twelve of whom are Doctors of Divinity, and numerous other characters of equal respectability."

In different parts of Europe it is said that the demand for these little pieces of metal was so great, that the mechanics could not manufacture them fast enough to supply the wants of the people ; and although the labor, together with the materials of which they were composed, was not really worth more than a few cents, they were greedily purchased at twenty-five dollars a pair, which was the regular price of the patentee. All benevolent females carried a pair in their pockets to use on persons who were not able to purchase them : poets courted the muses in recording and consecrating their many triumphs : royalty and nobility smiled upon them, and the clergy were industrious with their pens in writing lengthy articles, in order to spread their usefulness. One wrote in metaphoric strains as follows—"I have used the Tractors with success in several other cases in my own family, and although like Naaman the Syrian, I cannot tell why the waters of Jordan should be better than Abana and Pharpa, rivers of Damascus; yet, since experience has proved them so, no reasoning can change the opinion." Thus verifying the lines of Burns, that—

"Ev'n Ministers, they hae been kenn'd,
In holy rapture
A rousing whid to vend
And nail't wi' Scripture."

It were useless to enumerate the many evidences of the positive success of this practice; page upon page, and indeed volumnes might be filled up with examples of the kind. The number of cures reported to have been performed in a very short time, in Great Britain, amounted to 5,000; and the number estimated from the best data the friends of the system could gather, including those not regularly reported, amounted to one million and a half. The mass of the Medical Profession, comprehending the most enlightened and independent members, opposed the practice and pronounced it a delusion,—not, however, without first making experiments and ascertaining that wooden tractors (the patients not being aware of the deception), produced effects quite as sudden, and relief full as marked as when metallic ones were used. For their opposition they were accused of the basest motives and feelings, in terms like the following: "For very obvious reasons Medical men must never be expected to recommend the use of Perkinism. The Tractors must trust for their patronage to the enlightened and philanthropic out of the Profession, or to Medical men retired from practice, and who know of no other interest than the luxury of relieving the distressed." And again, in the shape of a query—"Will the Medical man who has spent much money and labor in the pursuit of the arcana of Physic, and on the exercise of which depends his support in life, proclaim the inefficiency of his art, and recommend a remedy to his patient which the most unlettered in society can employ as advantageously as himself?" Much offence was taken (as we see now the case with the friends of Homœopathy,) at any attempt to class this system of practice in the same category with those acknowledged delusions which had preceded it; in relation to which Perkins himself says—"The motives which must have impelled to this attempt at classing the *Metallic Practice* with the most paltry of empirical projects, are but too thinly veiled to escape detection.

Notwithstanding the great popularity of this system; its thousand vaunted cures; the brilliant hopes which it excited in the lovers of health and longevity, and its prevalence even in the present century, it has yet been buried so long in the tomb of its sisters, that I doubt whether many of you ever heard more of it than what has just been detailed. During the last summer I commissioned a friend, who was visiting the Eastern cities, to procure Dr. Perkins' book for me, who assured me that he had searched, faithfully, all the book-stores that he could find in three or four of the largest cities in the United States, without being able to find a man who had ever heard the subject mentioned before, until at last he met with one who laughed outright as he propounded his inquires, and, in explanation to my somewhat offended friend at this novel reply from a stranger, told him that he had once been a melancholic, and that his Physician had recommended to him the reading of this book, which he did very much to the exercise of his risible faculties; that his question had suddenly brought to his mind two very ludicrous subjects—Perkins' book and his disease. Since that I have made other efforts to procure the book, but have not been able to do so, and all that I know about the subject I have gathered from Reviews. Here allow me to acknowledge my indebtedness for most of the facts which I have obtained in relation to it, to that beautiful little book written by our distinguished countryman, Oliver Wendell Holmes, M. D., of Boston, entitled "Homœopathy and its Kindred Delusions."

It may here be mentioned to the credit of our Profession, that, although we oppose these systems of delusion during the days of their triumph and prosperity, yet when they have outlived the affections of their best friends, and are called upon to yield up the last lingering spark of vitality, then it is that the firm and faithful enemy who opposed their progress in life, steps forward, and magnanimously performs for them the last sad offices—sees them quietly entombed, and writes their brief history and epitaph. This is a duty always left us, and most cheerfully we perform it ; and, disclaiming any feelings of irreverence in the sacred figure, may we not say, if not "first at the cross," we are at least "last at the sepulchre."

